# Effects on stone cell development and lignin deposition in pears by different pollinators

**DOI:** 10.3389/fpls.2023.1093661

**Published:** 2023-02-08

**Authors:** Chongchong Yan, Nan Zhang, Chao Xu, Qing Jin, Yongjie Qi, Yongping Cai

**Affiliations:** ^1^ Anhui Academy of Agricultural Sciences, Hefei, Anhui, China; ^2^ Insititute of Horticulture, Anhui Academy of Agricultural Sciences, Key Laboratory of Horticultural Crop Germplasm innovation and Utilization (Co-construction by Ministry and Province), Hefei, Anhui, China; ^3^ School of Life Science, Anhui Agricultural University, Hefei, Anhui, China; ^4^ College of Health and Elderly, Anhui Vocational College of City Management, Hefei, Anhui, China

**Keywords:** 'Dangshan Su' pear, pollination, stone cell clusters, microscopy, ultramicroscopy

## Abstract

**Introduction:**

The pear pulp is formed by the development of the ovary wall, which is the somatic cell of the female parent, and its genetic traits are identical to those of the female parent, so that its phenotypic traits should also be identical to those of the female parent. However, the pulp quality of most pears, especially the stone cell clusters (SCCs) number and degree of polymerization (DP), were significantly affected by the paternal type. Stone cells are formed by the deposition of lignin in parenchymal cell (PC) walls. Studies on the effect of pollination on lignin deposition and stone cell formation in pear fruit have not been reported. Methods: In this study, 'Dangshan Su' (*P. bretschneideri* Rehd.) was selected as the mother tree, while 'Yali' (*P. bretschneideri* Rehd.) and 'Wonhwang' (*P. pyrifolia* Nakai.) were used as the father trees to perform cross-pollination. We investigated the effects of different parents on SCCs number and DP, and lignin deposition by microscopic and ultramicroscopic observation.

**Results and Discussion:**

The results showed that the formation of SCCs proceeds was consistent in DY and DW, but the SCC number and DP in DY were higher than that in DW. Ultramicroscopy revealed that the lignification process of DY and DW were all from corner to rest regions of the compound middle lamella and the secondary wall, with lignin particles deposited along the cellulose microfibrils. They were alternatively arranged until they filled up the whole cell cavity to culminate in the formation of stone cells. However, the compactness of the wall layer of cell wall was significantly higher in DY than in DW. We also found that the pit of stone cell was predominantly single pit pair, they transported degraded material from the PCs that were beginning to lignify out of the cells. Stone cell formation and lignin deposition in pollinated pear fruit from different parents were consistent, but the DP of SCCs and the compactness of the wall layer were higher in DY than that in DW. Therefore, DY SCC had a higher ability to resist the expansion pressure of PC.

## 1 Introduction

*Pyrus bretschneideri* cv. ‘Dangshan Su’ is a Chinese pear species widely grown in China and throughout Asia (Xu, 2009). Most pears are self-incompatible fruits with obvious xenia, and the fruit quality is affected by the source of pollination. The effect of pollination source can be seen in attributes such as fruit hardness and sugar, acid, vitamin C, and SCC contents (Cai et al., 2010; Cheng et al., 2020; Mumtaz et al., 2020). We previously pollinated ‘Dangshan Su’ with pollen from two different varieties of pears and found that pollen from different parents significantly affected the expression of microRNAs and proteins related to lignin metabolism in pear fruit (Cheng et al., 2017; Li et al., 2018), thus affecting lignin deposition, which in turn affected stone cell formation in pear fruit and ultimately the taste of pear fruit.

Stone cells, also known as sclereids, are responsible for affecting pear quality and can be present in an individual fruit or in groups of fruit; groups of stone cells are called SCCs (Jin et al., 2013). Pear stone cells are classified as short stone cells, which are sclerenchyma cells with high levels of lignin and cellulose (Cai et al., 2010; Yan et al., 2014). These stone cells differentiate from PCs after the secondary wall of PCs deposit on their primary wall (Cheng et al., 2019). Stone cells develop through secondary thickening of the cell wall and lignin deposition. Uneven thickening of the secondary wall causes single pit to start to form(Lian et al., 2020; Chukhchin et al., 2021). Lignin deposition similar to numerous discrete granules first occur at the corner of the middle lamella (ML) within various primary cell walls. Biosynthesis, transport, and deposition of lignin are closely associated with stone cell development and influence the formation of stone cells in pears(Tao et al., 2009; Cai et al., 2010). Lignification extends from the corner to the remaining regions of the ML and the secondary wall simultaneously in the xylem cells of Populus tomentosa Carr (Guo et al., 2015). Due to the lack of relevant studies, it remains unclear how lignin deposits in the stone cell wall and what contributes to its secondary thickening during the development of pear fruit.

Many pollens can be used to fertilize the ‘Dangshan Su’ in Dangshan, Anhui Province, China, such as ‘Wonhwang’ (P. pyrifolia Nakai), ‘Jingbaili’ (P. ussuriensis Maxim.), ‘bali’ (P. communis Bartlett), and ‘yali’ (P. bretschneideri Rehd) (Cheng et al., 2017). Since the two varieties, ‘Yali’ and ‘Wonhwang’, are also widely grown in Dangshan. Therefore, the local area mainly use them as the parent to pollinate the ‘Dangshan Su’. Thus, the pollen of ‘Yali’ and ‘Wonhwang’ was used to fertilize ‘Dangshan Su’ in this study. We compared the influence of these pollination types on the stone cell development and lignin deposition of ‘Dangshan Su’ fruits.

The results of this study can not only clarify the effects of pollination types on stone cell formation in pear, but also provide new insights into the mechanism of secondary wall formation of stone cells in pear. These results provide a theoretical foundation for selecting optimal pollination varieties for the ‘Dangshan Su’ pear, which could potentially improve the fruit quality.

## 2 Materials and methods

### 2.1 Materials

Fruits were obtained from pear trees grown on a farm (34°16’N 116°29’E) at the experimental site of the Institute of Horticulture, Anhui Academy of Agricultural Sciences, Dangshan County, Anhui Province, China. The ‘Dangshan Su’ female parent was fertilized with pollen from *P. bretschneideri.* ‘Yali’ (DY) and *P. pyrifolia.* ‘Wonhwang’ (DW). Buds on the short branches of ‘Dangshan Su’ with similar developmental stages and a similar size were selected from the mid-crown area on the south side of each tree. The stamens were removed from the buds and fertilized with the same amount of pollen from ‘Yali’ or ‘Wonhwang’. We also selected pollen from buds with similar developmental stages and similar sizes. The newly pollinated buds were covered with yellow single layer bags for seven days after pollination. Previous reports demonstrated that pear stone cell development occurs from 15 DAF to 67 DAF (Cai et al., 2010). Therefore, equally sized pear fruits were sampled starting from 15 DAF. A total of 9 developmental periods were sampled: 15 DAF, 23 DAF, 31 DAF, 39 DAF, 47 DAF, 55 DAF, 63 DAF, 87 DAF and the mature period (145 DAF). Samples of pear fruit with similar size were collected. The fruits were frozen and brought back to the lab. A part of the pear samples were fixed with FAA and 3.5% formaldehyde and then stored under low temperature. Stone cell development was observed by microscopy and lignin deposition was observed by ultra-microscopy.

### 2.2 Observation of stone cell clusters

The morphology of the SCCs was observed according to the method of Yan et al.(Yan et al., 2014). Transverse sections of pear fruits were manually prepared and stained with 1.0% phloroglucinol and 1.0 M hydrochloric acid as described in the Wiesner lignin staining method (Escamez et al., 2017). The size and distribution of SCCs were observed after chromogenesis.

### 2.3 Microscopy of stone cell development and lignin deposition

The pulp, between 2 mm inside of the peel and 0.5 cm outside of the stone, was cut with a sharp blade to the appropriate size. The pulp cubes were subsequently fixed with FAA fixative (50 ml of 50% ethanol, 5 ml of acetic acid, and 5 ml of formalin) and manually sliced. One drop of 1 M Hcl was placed onto the sample on a slide, which was then stained with a drop of 0.1% phloroglucinol/ethanol. To further examine the lignin deposition in the stone cell wall during the development of the pear fruit, sections were treated with the Wiesner reagent (phloroglucinol - HCl staining) and observed under an oil immersion microscope (× 630) (Clifford, 1974; Tao et al., 2009). A slide was microscopically examined, photographed and statistical stone cell mass size and distribution (Cai et al., 2010). The experiment was repeated six times, with three samples observed each time.

### 2.4 Scanning electron microscopy of stone cell distribution

Scanning electron microscopy (SEM) of stone cells was performed according to the method of Xu et al. (Xu et al., 2006). First, the fruit pulp sampled was screened from 2.0 mm under the peel to 0.5 mm outside the core was cut into small pieces (0.5 cm × 0.4 cm × 0.2 cm). The pulp was fixed for 1 h in 3.5% glutaraldehyde in phosphate buffer under low vacuum, followed by 2.5 hours at atmospheric pressure. Each sample was then fixed, washed and dried and 10 nm gold particles were plated onto the surface of the sample using a sputter coater. The samples were observed, photographed and content the stone cells number under the SEM (S-3000N, Hitachi, Japan) under high vacuum. The applied voltage between 6.00 kV 40 × and 6.00 kV 50 × and the working distance between 14.3 mm and 15.3 mm.

### 2.5 Ultramicroscopy of lignin deposition

Samples were stained with potassium permanganate (KMnO_4_) and examined using transmission electron microscopy (TEM). The pulp from 2 mm inside of the peel to 0.5 cm outside of the stone was cut with a sharp blade to generate blocks of 0.5 cm × 0.2 cm × 0.2 cm. The blocks were immediately placed into fixation solution containing 3.5% glutaraldehyde and 0.2 M phosphate (pH 7.2), fixed under low vacuum for 1 h, followed by atmospheric pressure for 2.5 h. The samples were then washed with phosphate buffer and fixed in 1% osmium tetroxide (in 0.2 M phosphate buffer, pH 7.2) at 4°C overnight. The samples were subsequently dehydrated in a series of Spurr resin-water mixtures (1:2, 1:1 and 2:1), supplied by Marsys-Tech Co. Ltd., (Beijing, China, catalog number S2690), before being treated twice with pure Spurr resin. They were then embedded and aggregated, and cut into ultrathin sections with an LKB-2188 microtome. The ultrathin sections were collected with a 100 - mesh copper net. Some of the sections were double stained with uranyl acetate and lead citrate. The remaining sections were stained with 1% KMnO_4_ solution (in 0.1% sodium citrate) for 3 min (Yin et al., 2003; Day et al., 2005). The stained sections were examined and photographed under a TEM (Philips EM-400T). The experiment was repeated six times, with one sample observed each time.

## 3 Results

### 3.1 Stone cell clusters distribution and number in DY and DW

After the Wiesner reaction, it could be revealed from the fruit sections that pear SSCs were stained rose red, in contrast to their surrounding PCs that were barely stained (**Figures A1, B1**). SSCs and PCs were arranged in a mosaic pattern such that SSCs were embedded in PCs (Shutian Tao et al., 2009). However, SSCs were not evenly distributed among PCs, they concentrated radially near the fruit center. The distribution of SCCs near the fruit center was also significantly more intensive than that in other areas. At the same time, we found that the nuclei of DY were smaller than DW, but the density and distribution range of SCCs were higher than that of DW. Observation of DY and DW using SEM, we can found that the unit vision of DY showed that the number of the SCC was five, whereas in the DW it was only four, which also suggested that the distribution of SCCs was more intensive in DY than in DW (**Figures A2, B2**). This result is consistent with the results obtained from Wiesner reaction. It showed that pollination by different parents could change the distribution of SCCs in pear fruit.

**Figure 1:**
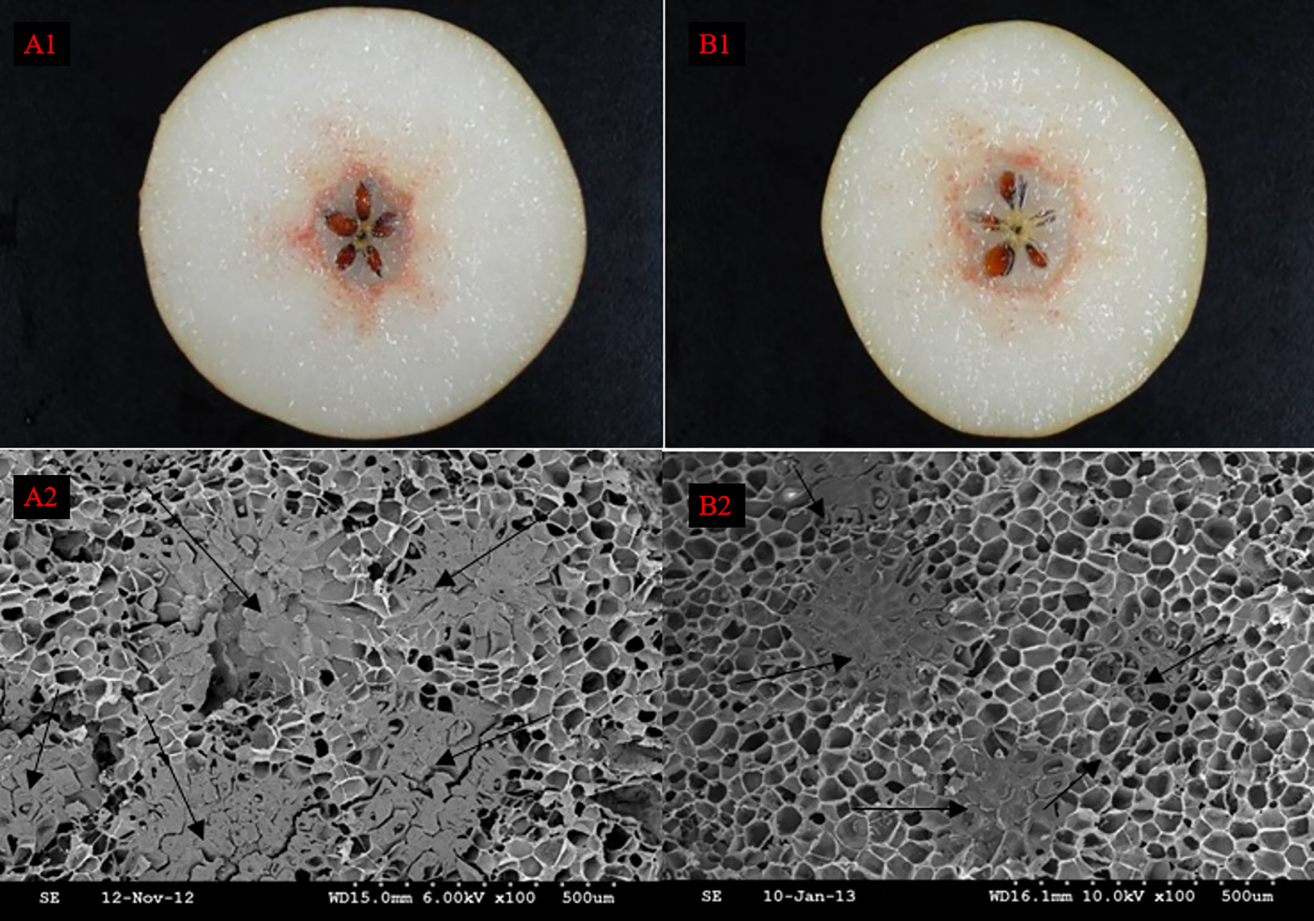
The distribution of stone cell cluster of DY and DW. **A1** Cross section staining of DY, **A2** Scanning electron microscopy of DY, **B1** Cross section staining of DW, **B2** Scanning electron microscopy of DW.

### 3.2 Comparison the number and area of stone cell clusters in DY and DW

**Figure 2:**
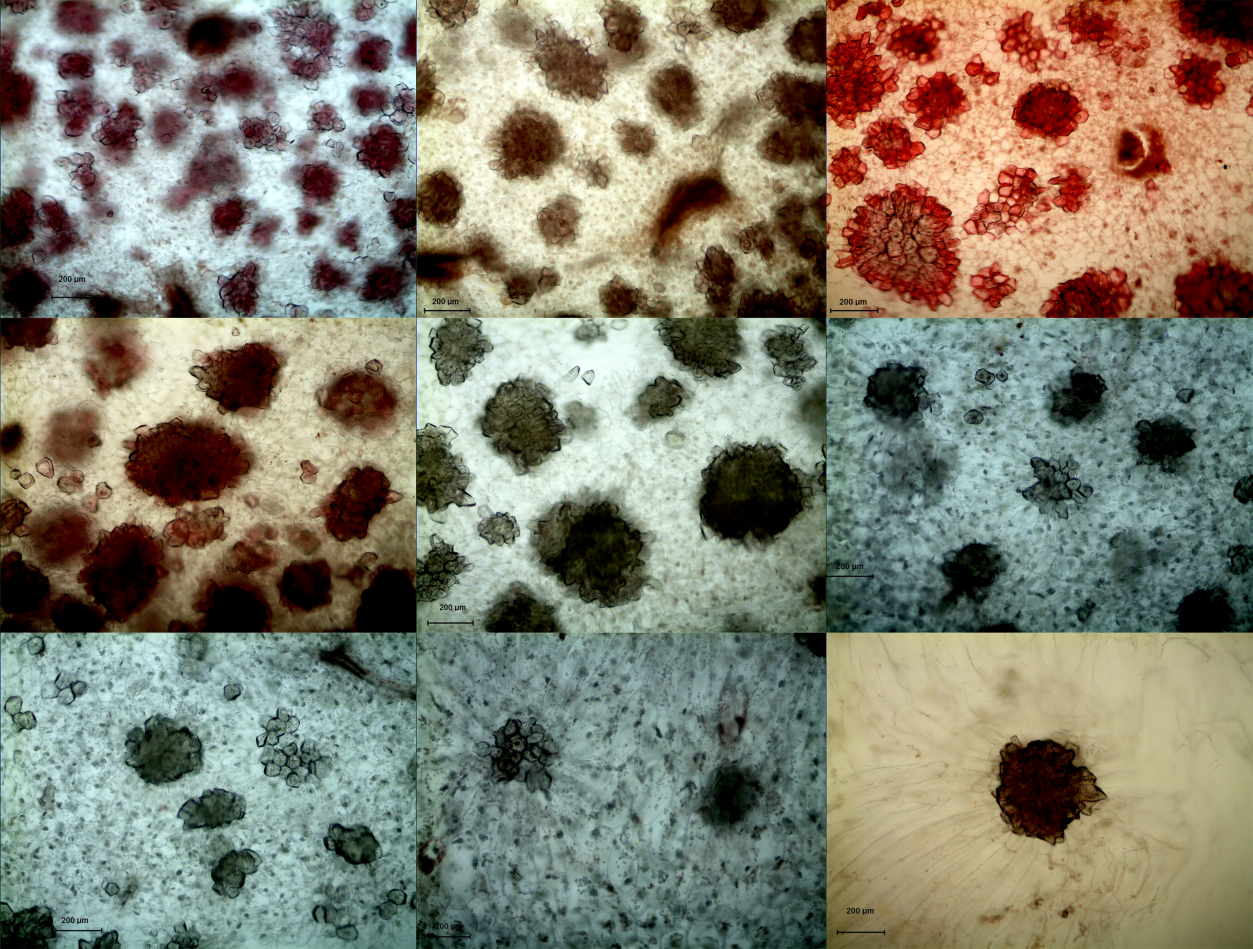
Microscopy of stone cell cluster development in DY(100×) A - I: 15d、23d、31d、39d、47d、55d、63d、87d、145d, respectively.

**Figure 3:**
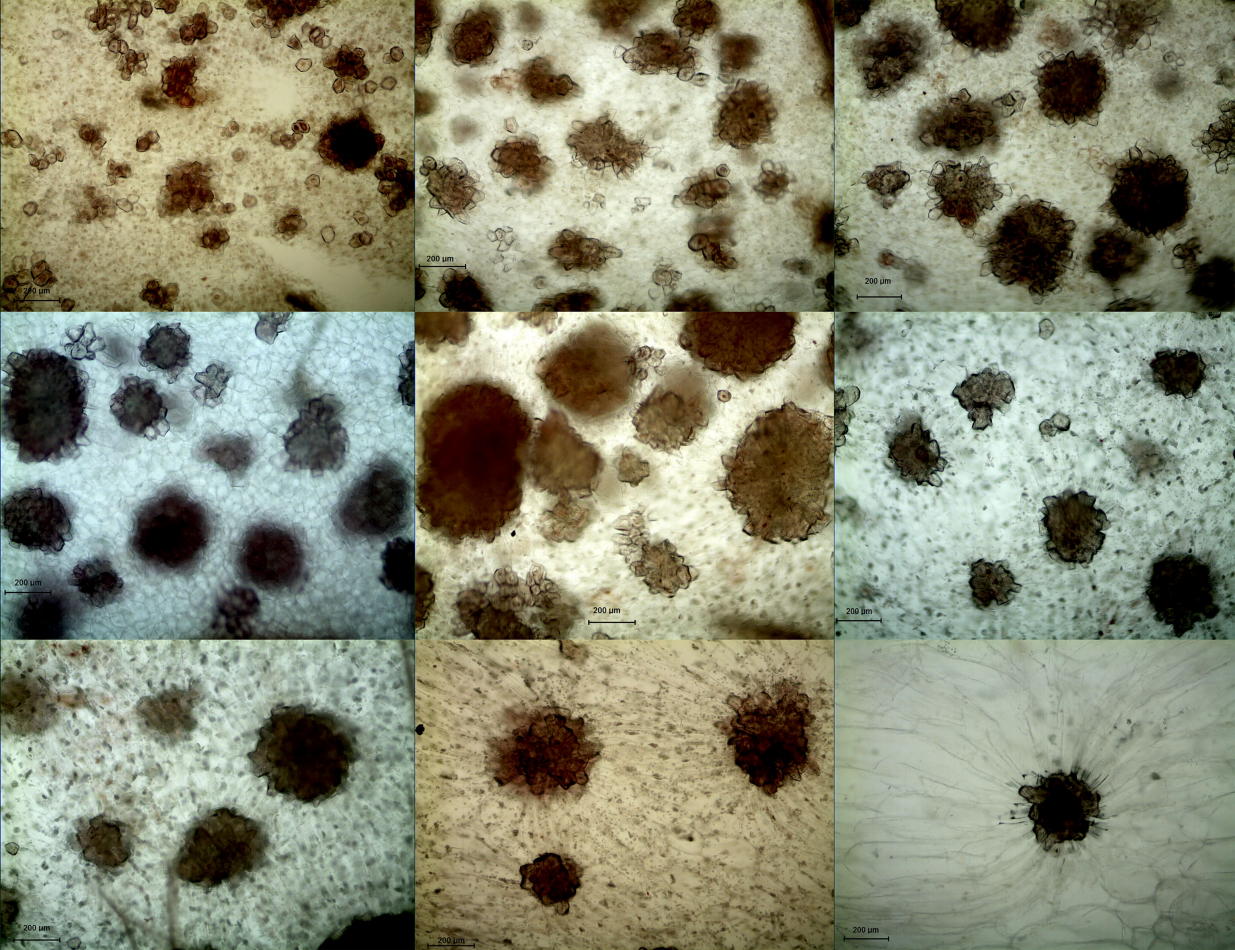
Microscopy of stone cell cluster development in DW (100 ×) A - I: 15d、23d、31d、39d、47d、55d、63d、87d、145d, respectively.

In both DY and DW, the area of SCCs initially increased and subsequently decreased over the course of fruit development (**Figures 2**, **3**). At 23 DAF, the major SCCs in the fruits of DY and DW were in the primitive stage with loose stone cell DP. The SCC area of the DY and DW increased rapidly from 23 to 55 DAF. From 23 to 55 DAF, the SCC area of DY and DW increased rapidly, from 0.02 mm^2^ to nearly 0.1 mm^2^, and the formation of SCCs was largely complete at 55 DAF, after which it slowly decreased to about 0.06 mm^2^. The number of SCCs was highest from 23 to 31 DAF and then gradually decreased. This might be due to the small size of pear fruit in the early stage of fruit development, and the number of SCCs in the unit field of view was more. With the development, the volume of fruit continues to increase, which leaded to the decrease of SCCs in the unit field of view. This was consistent with the findings of Lee et al. (Lee et al., 2006; Nie et al., 2009).

**Figure 4:**
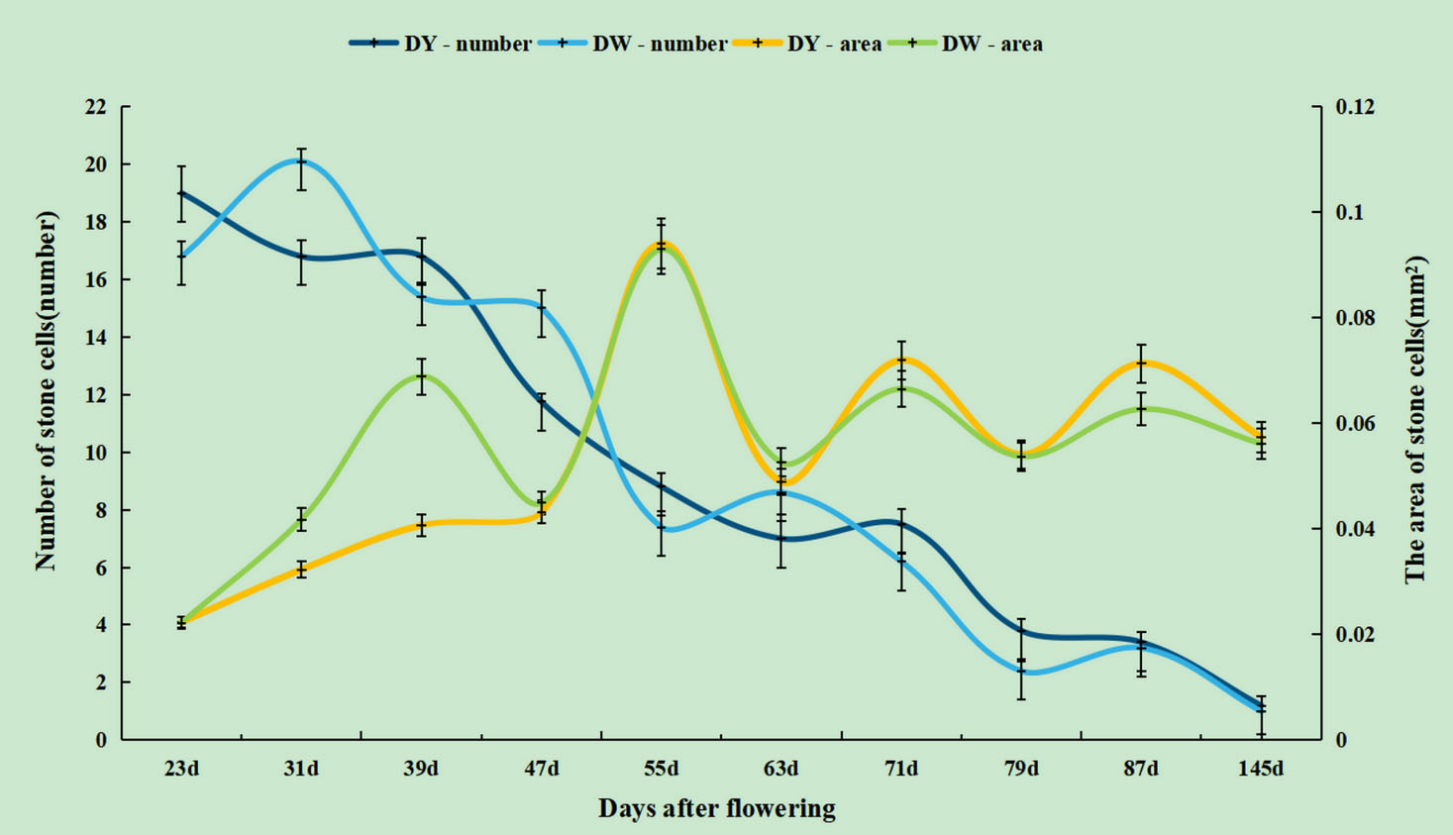
The number and area of stone cell clusters during different developmental stages of DY and DW.

During pear fruit development, the number of SCCs in DW was higher than that in DY only at 31, 47 and 63 DAF. The area of DW SCCs was higher than that of DY only in 31, 39, 47 and 63 DAF (**Figure 4**). It could be seen that when the area of SCCs was higher in DY, the number of SCCs was also higher. The results indicated that the influence of pollen sensation on the number and area of SCCs in pear fruit was spatiotemporal.

### 3.3 Comparison of lignin deposition and stone cell development in DY and DW

Lignin deposition and transport were closely related to lignification of stone cells (Jin et al., 2013). The cell wall of pear fruit stone cells were generally divided into middle Lamella (ML), primary wall (PCW) and secondary wall (S). The secondary wall was divided into outer secondary wall (S1), middle secondary wall (S2) and inner secondary wall (S3). The composite middle lamella (CML) was formed between the middle lamella and the primary wall (Tao et al., 2009). To further investigate the effect of pollination by different parents on the deposition of lignin on stone cells in Dangshan su, TEM was used to observe DY and DW sections at different stages after flowering. **Figure 6** clearly showed the lignin deposition on stone cells at different developmental stages. It could be seen that the lignin deposition process on stone cells of DY and DW was no significant difference.

**Figure 5:**
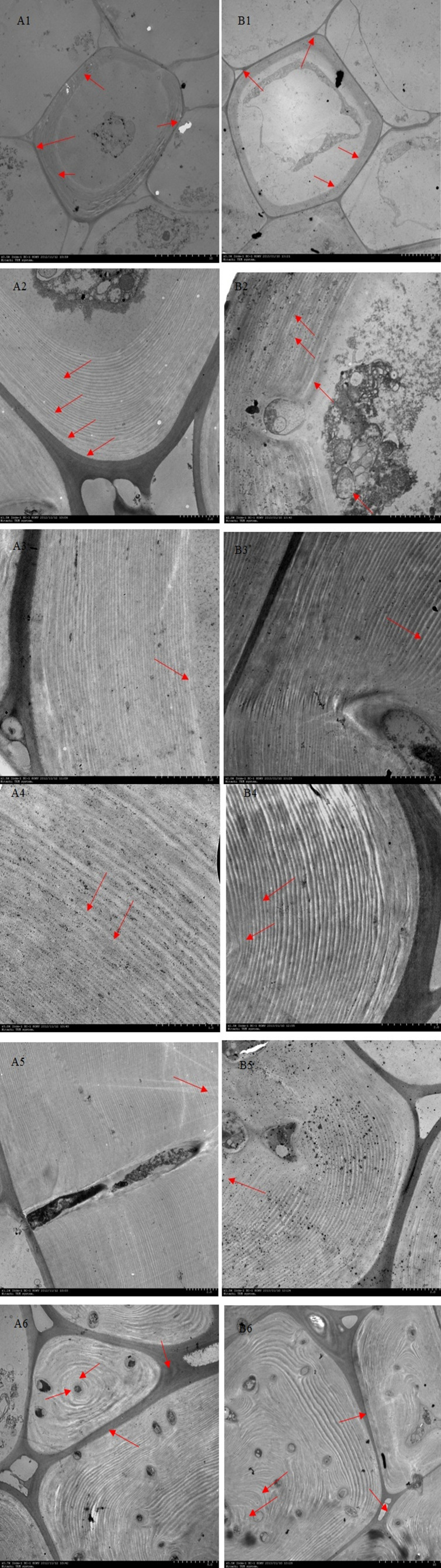
Ultramicroscopy of lignin deposition during stone cell development of DY and DW. Note: **(A1 – A6)** 23d, 31d, 39d, 47d, 55 d and 63 days after flowering of DY, respectively; **(B1 – B6)** 23d, 31d, 39d, 47d, 55d and 63 days after flowering of DW, respectively. **(A1,B1)** Lignin was unevenly deposited along the primary walls fine particles at the corner of the primary cell wall; **(A2-A3,B2-B3)** Deposition initiates from the inside of the S1 layer to S2 layer; **(A4-A5,B4-B5)** Lignin particles were deposited unevenly along the inside of each micro-fibril in every S_2_ layer; **(A6,B6)**Lignin was fully occupied the cell cavity; CC: cell corner; CIL: composite intercellular layer; S_1_: S_1_ layer of the secondary wall; S_1_L: layer between S_1_ and S_2_; S_2_: S_2_ layer of the secondary wall; S_2_L: layer between S_2_ and S_3_; S_3_: S_3:_ layer of the secondary wall.

The process of lignin deposition and stone cell development was generally as follows: 1. 23 DAF, lignin was deposited from the corner of cell (CC) in dispersed strip granules. With deposition of cellulose microfibrils (CMfs) along the perimeter of cell and oriented arrangement, lignification process began to expand along the CC of primary wall to other regions of CML and S layer (**Figures 5A1, B1**); 2. 31-39 DAF, granular lignin was in homogeneous deposited on S_1_ layer oriented CMfs, starting from the inner side of S_1_ (**Figures 5A2-A3, B2-B3**); 3. When the fruit developed to 47-55 DAF, S_2_ CMfs were also arranged along the circumference of the cell and stratified. At this time, S_1_ and S_2_ had a relatively obvious junction (S_1_L), where the staining was deep, indicating that there was more lignin deposition. Lignin particles were inhomogeneous deposited along the CMfs at S_1_L, and lignin deposition started from the CMfs in the inner layer of S_2_ (**Figures 5A4-A5, B4-B5**); 4. As lignification proceeded, the CMfs of the secondary wall S_3_ remain oriented along the circumference of the cell and the lignin particles continue to be deposited unevenly along the CMfs. As lignin particles began to deposit in S_3_, lignin was rapidly deposited throughout the secondary wall, forming alternating light and dark streaks, with the light streaks being CMfs and the dark streaks being lignin-deposited particles, until they filled the whole cell lumen (As shown by the short arrows in A6, B6).

**Figure 6:**
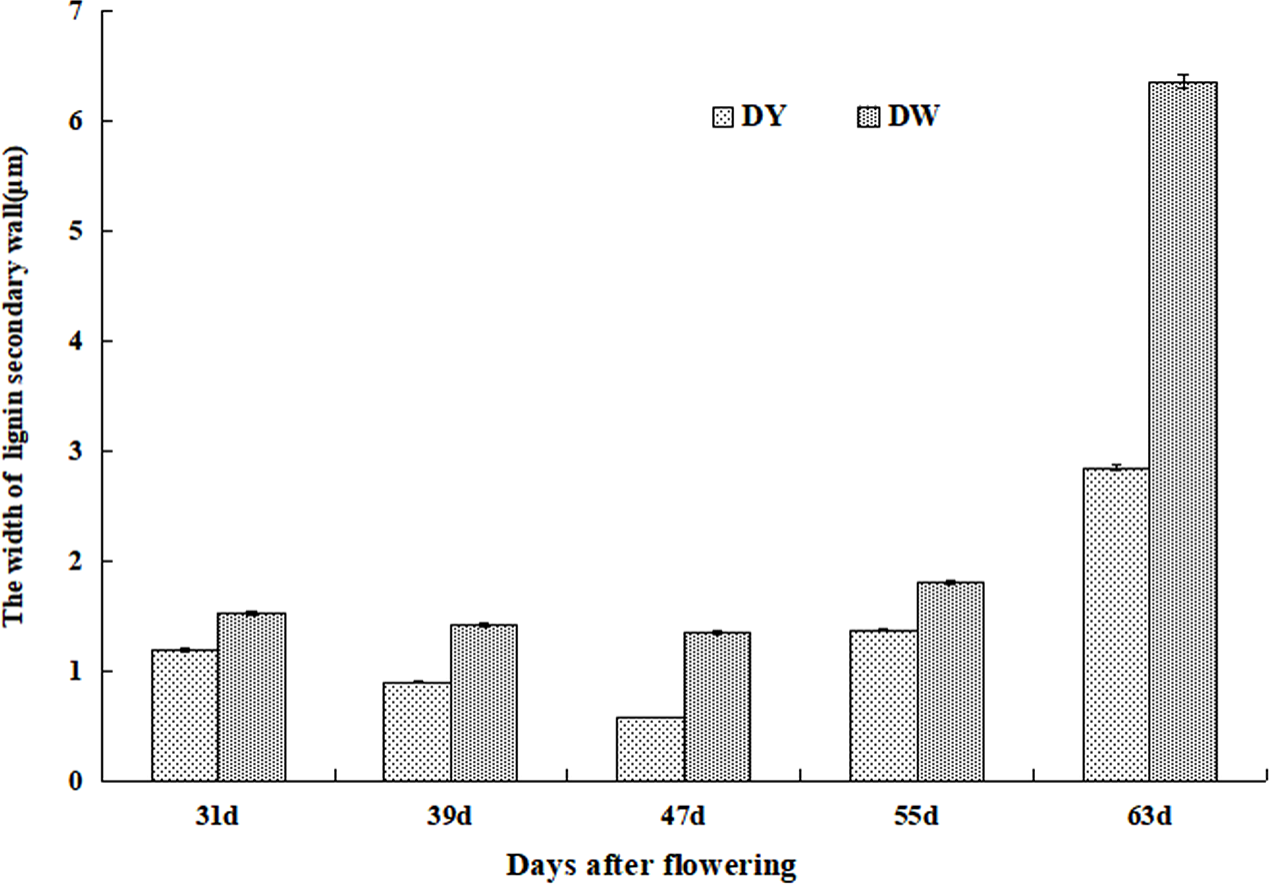
The width of Secondary wall layer of DY and DW.

Although the development processes of DY and DW stone cells were basically the same, there were differences in the compactness of the secondary wall of stone cells in different developmental stages. The compactness of the secondary wall of DY lignin deposition was higher than that of DW. Statistical analysis of the micrographs of lignin deposition at different developmental stages of DY and DW showed that the width of the secondary parietal layer of lignin deposition in DW fruits was larger than that in DY at all developmental stages, especially at 47 DAF, where the former was 2.34 times greater than the latter (**Figure 6**). This may be due to the different ratios of lignin G/S monomers and bond structures in pear fruit after pollination by different parents. At the same time, due to the different compactness of lignin deposition in the secondary parietal layer, the resistance of the SSCs formed by PCs to the swelling pressure caused by PCs expansion was different, which might explain the different changes in the area of SCCs in pears pollinated by different parents during late fruit development. This might also be one of the important factors causing the difference in the DP of DY and DW SCCs.

### 3.4 Ultramicroscopic observation of stone cell pit in pear

**Figure 7:**
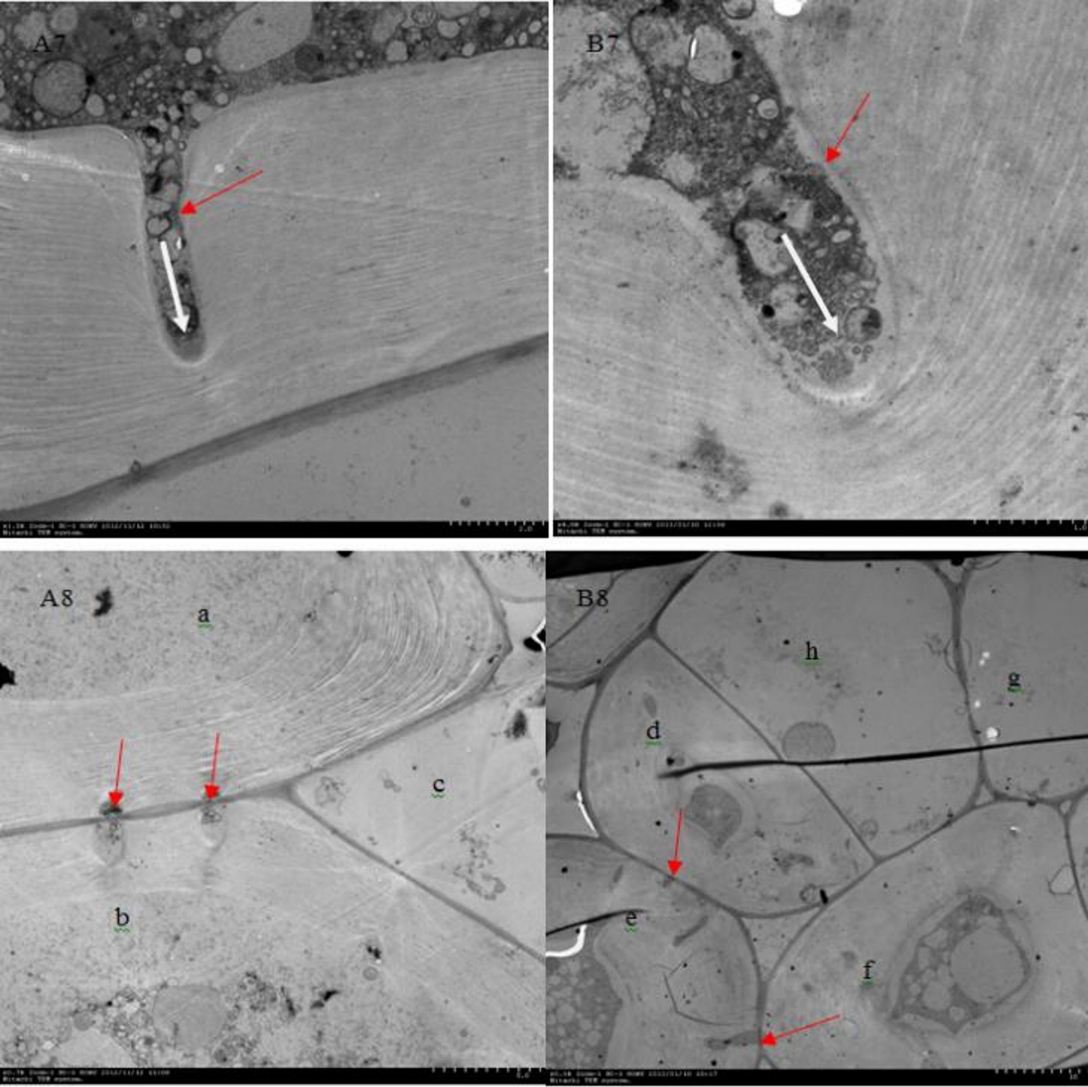
Ultramicroscopy of pits on pear stone cells of DY and DW. **(A7, A8)** was DY, **(B7, B8)** was DW. **(A7, B7)** Pits on the stone cells (short red arrow), the direction of material transport was from the intracellular to extracellular (short white arrow). **(A8)**’a’ cell and ‘b’ cell were lignified parenchyma cells, ‘c’ cell was still parenchyma cell. There were two pit pairs between the ‘a’ cell and ‘b’ cell (short red arrow), but no pit pair between ‘a’ cell and ‘c’ cell or between ‘b’ cell and ‘c’ cell. **(B8)** ‘d’ cell, ‘e’ cell and ‘f’ cell were lignified parenchyma cells, ‘h’ cell an ‘g’ cell were just parenchyma cells. There was pit pairs between ‘d’ cell and ‘e’ cell as well as between ‘e’ cell and ‘f’ cell (short red arrow), but no pit pairs between ‘d’ cell and ‘g’ cell or between ‘d’ cell and ‘h’ cell or between ‘f’ cell.

The structural units of the primary and secondary walls of plant cells were mainly bundles of cellulose molecules (Lopez and Barclay, 2017). These bundles of cellulose molecules were bonded in layers by hydrogen bonds at certain angles and directions. The thin parts that form at certain points were called pits. The protoplasmic filaments that connected two adjacent cells to each other through the stria were called plasmodesmata, which were the bridge between cells for direct material and information contact (Sasaki et al., 2017; Chukhchin et al., 2021). In order to further investigated the changes in pits during lignification of stone cells and the effect of pits on the initiation of lignin processes in parenchymal cells, we carried out ultramicroscopic observations of the changes in pits in stone cells of DY and DW (**Figure 7**).

As can be seen from A7 and B7, the pits of stone cells in pear fruit are mainly single pits, and two adjacent cell pits are connected by plasmodesmata in the primary cell wall. There was a large and strong presence of highly electron-dense material in the area around the pits and within the pits. By the direction of transport of these high electron-dense materials, it can be assumed that the pits on pear stone cells mainly transports degraded material from PCs that are beginning to lignify out of the cell, unlike the role of the pits in other plants, which mainly provides a water transport channel for cells with secondary walls (Sano et al., 2011).

As can be seen from A8 and B8, the PCs surrounding the stone cells also began to lignify if they were connected to the stone cells by pits, whereas the PCs not connected to the stone cells by pits did not lignify. As in A8, both ‘a’ and ‘b’ cells were PCs that were beginning to lignify, but ‘c’ cell was still PC. There were two pairs of pits connected between ‘a’ and ‘b’ (indicated by red arrows), but in ‘a’ and ‘c ‘ and ‘b’ and ‘c’ were not connected by any pits. Also in B8, ‘d’, ‘e’ and ‘f’ cells were lignified PCs, while ‘h ‘ cells and ‘g’ cells were PCs that had not begun to lignify. Pit pairs were connected between ‘d’ and ‘e’ and between ‘e’ and ‘f’ (red arrows indicated by), but there were no pits between ‘d’ and ‘h’, ‘f’ and ‘h’ or no pairs were found between ‘d’ and ‘h’, ‘f’ and ‘h’, or ‘f’ and ‘g’. From this it could be seen that PCs surrounding a lignified PC, as long as there was a pits connection with the lignified PC, that PC also began to lignify to form a stone cell primordium. However, the PCs that were not connected to the PCs at the beginning of lignification were still PCs without lignification. Thus, it could be speculated that lignified PCs transported some signaling molecules that could initiate ligninization process of PCs to neighboring PCs through the pits, thus initiating lignification process of surrounding PCs. However, what such signaling molecules are remains to be further studied.

## 4 Discussion

Wiesner staining of cross-sections of DY and DW pear fruit, combined with SEM, revealed that at 47 - 63 DAF, DW had more SCCs than DY and fewer than DY at other times. The change trend of the DP of SCCs increased first and then decreased, reaching the maximum value at 55 DAF. The trends in the DP and number of SCCs in DY and DW were basically the same, but there were differences in content, indicating that pollination by different parents mainly affected the DP and number of SCCs in pear fruit, but did not change the stone cell development process. Therefore, it could be speculated that the number and DP of SCCs in pear fruit were quantitative traits controlled by multiple genes, which was consistent with the results of previous studies(Zhang et al., 2021).

The SCC was a group of lignified PCs, and how the “lignified” PCs “shrink” had been postulated to be due to the breakdown of the large SCCs into individual SCCs by laccase, pectinase and cellulase, which in turn were degraded by enzymes, resulting in a reduction in the DP of the SCCs at a later stage of development (Xue et al., 2019; Wang et al., 2020). However, Lee et al. suggested that the reduction in the number and DP of SCCs in late development was not due to enzymatic breakdown, but rather to the fact that SCCs form slower than the rate of fruit expansion and are ‘squeezed and diluted’ by PCs (Lee et al., 2006).

The stone cell development and lignin deposition at different developmental stages of DY and DW were observed by TEM and found that the processes were largely consistent. However, there were significant differences in the compactness of the lignin-deposited secondary wall wall layers, with those of DW significantly wider than those of DY. In the studies of bamboo and wood, they were found that the compactness the secondary parietal layer, the higher the stability and compressive resistance (Murphy and Alvin, 1997; Suzuki and Itoh, 2001). Therefore, it was speculated that at the late stage of fruit development, due to the different pollination fathers, the compactness of the secondary wall of stone cells was different, which led to the different ability of the stone cell to resist the expansion pressure of PCs, which led to the different changes in the SCCs DP of DY and DW, and thus affecting the DP of SCCs.

In this study, we found that the pit was mainly in simple pit pair, with the pit pairs of adjacent cells connected by plasmodesmata at the primary cell wall, which is consistent with the type of pits and the way they are connected in the cell walls of plants such as Wheat, Eucommia and Mao bamboo (Kristensen et al., 2008; Fromm, 2013; Lian et al., 2019). By analyzing the direction of transport of highly electron-dense material within the pits, it was hypothesized that the role of the pits on pear stone cells was to transport degraded material out of the cell from PCs which were beginning to lignify, and that the transported materials might contain some kind of signalling molecules that could initiate the process of lignification in PCs, thus accelerating the process of lignification in PCs surrounding the stone cells. It had been speculated that the substance in pear stone cells may be calcium ions (Lee et al., 2006; Tao et al., 2009), and it had also been speculated in moso bamboo and wood that the signaling molecules may be H_2_O_2_ (Lian et al., 2020), but neither could give sufficient evidence, so the specific substance of the signaling molecules need to be further studied.

Therefore, a better understanding of the effects of pollination by different parents on pear stone cell development and lignin deposition. It can provide a scientific basis for clarifying the effect of different parental pollination on pear stone cell development, and thus having great significance for pear breeding.

## 5 Conclusion

**Figure 8:**
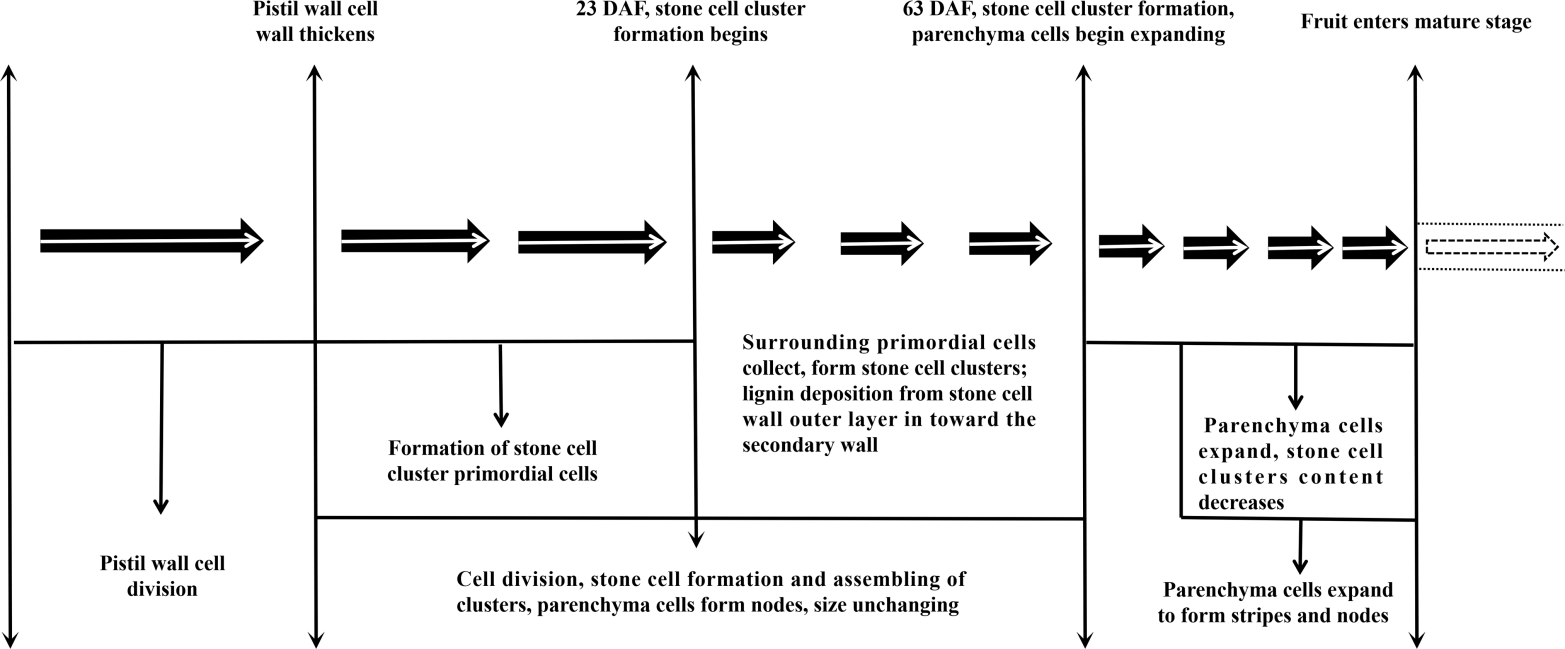
Stone cell Development in pear.

This study proved that the formation process of DY and DW SCC was consistent, but the SCC number and DP in DY was higher than that in DW. All of the ultramicroscopy results regarding stone cell development and lignin deposition in Dangshan Su pears are summarized in **Figure 8**: The lignification process of DY and DW were all from corner to rest regions of the compound middle lamella and the secondary wall, with lignin particles deposited along the cellulose microfibrils. They were alternatively arranged until they filled up the whole cell cavity to culminate in the formation of stone cells. However, the compactness of the wall layer of cell wall DY was significantly higher than that of cell wall DW. Therefore, DY SCC had a higher ability to resist the expansion pressure of PC. We also found that the pit of stone cells was predominantly single pit pair, they transported degraded material from the PC that were beginning to lignify out of the cells.

## Data availability statement

The original contributions presented in the study are included in the article/supplementary material, further inquiries can be directed to the corresponding author/s.

## Author contributions

CY and NZ performed the experiments and wrote the paper. CX contributed reagents/materials/analysis tools. CY, NZ and QJ analyzed the data. CY, YC and YQ discussed and analyzed the results. YC conceived and designed the experiments. All authors read and approved the final manuscript. CY and NZ have contributed equally to this work. All authors contributed to the article and approved the submitted version.
